# Evaluation of the Chemical and Antioxidant Properties of Wild and Cultivated Mushrooms of Ghana

**DOI:** 10.3390/molecules191219532

**Published:** 2014-11-26

**Authors:** Mary Obodai, Isabel C.F.R. Ferreira, Ângela Fernandes, Lillian Barros, Deborah L. Narh Mensah, Matilda Dzomeku, Arailde F. Urben, Juanita Prempeh, Richard K. Takli

**Affiliations:** 1 CSIR-Food Research Institute, P.O. Box M20, Accra, Ghana; E-Mails: obodaime@yahoo.com (M.O.); lnarh@yahoo.com (D.L.N.M.); matildadela@yahoo.com (M.D.); Juanita_luv06@yahoo.com (J.P.); rtakli@yahoo.com (R.K.T.); 2 Mountain Research Centre (CIMO), ESA, Polytechnic Institute of Bragança, Campus de Santa Apolónia, apartado 1172, Bragança 5301-855, Portugal; E-Mail: afeitor@ipb.pt; 3 Embrapa Recursos Genéticos e Biotecnologia—PqEB—Parque Estação Biológica, Final Av. W5 Norte, Caixa Postal 02372, CEP:70770-900 Brasília, DF, Brazil; E-Mail: arailde.urben@embrapa.br

**Keywords:** wild mushrooms, nutritional value, antioxidant potential, cultivated mushrooms, macro- and micro-elements

## Abstract

Knowledge of the chemical composition of both wild and cultivated edible mushrooms in Ghana is limited. This study reports their nutritional value, composition in lipophilic and hydrophilic molecules, minerals and antioxidant properties. The samples were found to be nutritionally rich in carbohydrates, ranging from 64.14 ± 0.93 g in *Pleurotus ostreatus* strain EM-1 to 80.17 ± 0.34 g in *Lentinus squarrosulus* strain LSF. The highest level of proteins (28.40 ± 0.86 g) was recorded in the mentioned *P. ostreatus* strain. Low fat contents were registered in the samples, with *Auricularia auricula* recording the lowest value. High levels of potassium were also observed with the following decreasing order of elements: K > P ~ Na > Mg > Ca. High levels of antioxidants were also observed, thus making mushrooms suitable to be used as functional foods or nutraceutical sources. Furthermore, this study provides new information regarding chemical properties of mushrooms from Ghana, which is very important for the biodiversity characterization of this country.

## 1. Introduction

Wild mushrooms are considered a popular delicacy in several countries all over the world and are collected and consumed when in season. In Ghana, these wild mushrooms are collected in early March to April and later on in September to October from the forests in the rural areas and subsequently sold at the urban centers. This is an old tradition and a well-established activity, which is gender-related and generally regarded as work for women and children [1].

Wild mushrooms are rich in minerals and have high levels of water, proteins, fibers and carbohydrates [2]. The two most commonly preferred wild edible mushroom species collected in Ghana are *Termitomyces* and *Volvariella* spp. These mushrooms are preferred for different reasons such as taste, attractiveness, uses as substitutes for meat or fish, aroma and medicinal values [3]. The genus *Termitomyces*, which is the most preferred and cherished in the country and in many African and Asian countries such as Zambia, Tanzania, Burundi, Thailand and Taiwan, cannot be currently cultivated as its cultivation is difficult because of the very specific condition under which it grows in Nature. It grows naturally, in symbiotic association with termites known as “fungus growers” (Macrotermitinae), which grow about 30 species of this genus worldwide [4]. Nutritionally, *Termitomyces* sp. have been found to be higher in protein content than other edible mushrooms in Nigeria and Malawi [5,6]. The most common *Termitomyces* species recorded in Ghana are *Termitomyces globulus*, *T. schimperi*, *T. robustus*, *T. reticulates*, *T*. *microcarpus* and *T. clypeatus*, and these have a wide range of medicinal uses such as blood tonic, for malnourished children suffering from kwarshiokor (a protein deficiency condition), and also in the treatment of rheumatism and diarrhea [7].

Mushrooms have been reported as therapeutic foods useful in preventing diseases and they exhibit varied biological properties such as antibacterial, antimutagenic, antitumoral and antiviral activities [8,9]. These functional characteristics are mainly due to their chemical composition [10]. Traditionally, in Ghana rural people consume mushrooms also for medicinal reasons such as reducing obesity and lowering blood pressure in hypertensive patients, among others. *Pleurotus tuber-regium* has been used by traditional herbal doctors for increasing the weight of underweight children, treating asthma and high blood pressure, among others [11].

Since 1990, cultivation of mushrooms in Ghana, has mainly been for oyster mushrooms such as *Pleurotus ostreatus*, *P. sajor-caju*, and woodear mushrooms (*Auricularia* spp.) and quite recently, on an experimental basis, *Lentinus squarrosulus*. These have been cultivated on composted sawdust of *Triplochiton scleroxylon* and or mixtures with *Chlorophora excelsa*.

Little knowledge about these cultivated mushrooms in terms of chemical composition and antioxidant properties is available. Therefore, in the present study five cultivated and two wild mushroom samples were evaluated for their nutritional value, composition in lipophilic (saturated and unsaturated fatty acids) and hydrophilic (free sugars, organic acids and phenolic compounds) molecules, minerals (macro- and micro-elements), and antioxidant properties (free radical scavenging activity, reducing power and lipid peroxidation inhibition) to ascertain their values for the nutraceutical and food industries.

## 2. Results and Discussion

### 2.1. Nutritional Value

The nutritional value and energetic contribution of the studied edible mushrooms are shown in Table 1. Almost all species gave similar ash contents, except *A. auricula*, and the result for this species is in agreement with the literature [12], and it gave the lowest content in fat, but the highest energetic contribution, due to the highest protein levels. The total fat, protein and ash values obtained for *P. tuber-regium* in this work, is also in agreement with the values reported by Gbolagade *et al.* [13]. Nevertheless, the total fat found in *P. sajor-caju* and *P. ostreatus* is lower than the values reported for samples cultivated on banana and rice straw, respectively, 5.26 and 4.99 g/100 g for *P. sajor-caju*, and 5.97 and 6.32 g/100 g for *P. ostreatus* [14].

Concerning protein content, *P. ostreatus* gave the highest value. The values available in the literature are variable: 19.93 to 34.73 g/100 g for samples cultivated on wheat straw supplemented with sugar beet [15]; 21 g/100 g in *P. ostreatus* cultivated on wheat straw [16] and 9.62 g/100 g in a sample cultivated on coffee husks [17]; the results obtained in this work are similar to those reported for a wild sample from Croatia [18].

The fatty acids composition, total saturated fatty acids (SFA), monounsaturated fatty acids (MUFA) and polyunsaturated fatty acids (PUFA) results are shown in Table 1. Twenty-four fatty acids were quantified in *L. squarrosulus* strain LSF and *P. ostreatus*, and twenty-three in *P. tuber-regium*, *T. robustus*, *L. squarrosulus* strain SQW, *P. sajor-caju* and *A. auricula*. A prevalence of PUFA over SFA was observed, except for *P. tuber-regium*, where SFA were dominant. The most abundant fatty acid in all the samples was linoleic acid (C18:2n6). The second most abundant fatty acid in *P. tuber-regium*, *P. ostreatus*,*P. sajor-caju* and *A. auricula* was oleic acid (C18:1n9), while in *T. robustus*, *L. squarrosulus* strain SQW and *L. squarrosulus* strain LSF it was palmitic acid (C16:0). The global percentages obtained for SFA, MUFA and PUFA in *P. ostreatus* and *P. sajor-caju* are very similar to those presented in other studies [19,20].

### 2.2. Lipophilic and Hydrophilic Compounds

The results obtained for hydrophilic compounds (free sugars, organic acids and phenolic acids) are presented in Table 2. Trehalose was the most abundant sugar in all the samples; the highest level was found in *L. squarrosulus* strain LSF and *P. ostreatus*, while sucrose was only detected in *A. auricula* and fructose in *L. squarrosulus* strain LSF, *P. ostreatus* strain EM1 and *P. sajor-caju* strain PScW.

**Table 1:** Nutritional value and lipophilic compounds of wild and cultivated mushrooms of Ghana (mean ± SD).

	*Pleurotus tuber-regium*	*Termitomyces robustus*	*Lentinus squarrosulus* strain SQW	*Lentinus squarrosulus* strain LSF	*Pleurotus ostreatus* strain EM-1	*Pleurotus sajor-caju* strain PScW	*Auricularia auricula*
Nutritional value							
Fat (g/100 g)	1.30 ± 0.03 ^e^	1.42 ± 0.01 ^de^	1.98 ± 0.18 ^b^	1.72 ± 0.05 ^c^	1.49 ± 0.01 ^d^	2.93 ± 0.07 ^a^	0.82 ± 0.03 ^f^
Protein (g/100 g)	13.31 ± 0.10 ^d^	14.78 ± 0.07 ^c^	19.43 ± 0.76 ^b^	12.51 ± 0.36 ^d^	28.40 ± 0.86 ^a^	15.33 ± 0.45 ^c^	19.27 ± 0.80 ^b^
Ash (g/100 g)	6.29 ± 0.18 ^a^	6.21 ± 0.45 ^a^	6.13 ± 0.24 ^ab^	5.60 ± 0.11 ^b^	5.96 ± 0.37 ^ab^	6.38 ± 0.09 ^a^	3.05 ± 0.43 ^c^
Carbohydrates (g/100 g)	79.10 ± 0.22 ^bc^	77.60 ± 0.34 ^c^	72.45 ± 0.88 ^d^	80.17 ± 0.34 ^b^	64.14 ± 0.93 ^e^	75.36 ± 0.38 ^d^	76.86 ± 0.63 ^a^
Energetic value (Kcal/100 g)	381.34 ± 0.41 ^f^	382.26 ± 1.31 ^ef^	385.39 ± 0.02 ^cd^	386.16 ± 0.50 ^c^	383.64 ± 1.08 ^de^	389.15 ± 0.02 ^b^	391.88 ± 1.11 ^a^
Lipophilic compounds							
C6:0	0.11 ± 0.03	0.22 ± 0.05	0.12 ± 0.01	0.06 ± 0.00	0.06 ± 0.02	0.05 ± 0.01	0.07 ± 0.01
C8:0	0.10 ± 0.02	0.07 ± 0.00	0.06 ± 0.02	0.06 ± 0.00	0.04 ± 0.00	0.03 ± 0.01	0.07 ± 0.02
C10:0	0.11 ± 0.02	0.06 ± 0.01	0.08 ± 0.01	0.04 ± 0.00	0.05 ± 0.01	0.04 ± 0.01	0.05 ± 0.01
C12:0	0.29 ± 0.05	0.11 ± 0.00	0.15 ± 0.01	0.09 ± 0.02	0.07 ± 0.00	0.09 ± 0.01	0.11 ± 0.02
C14:0	1.12 ± 0.07	0.91 ± 0.03	0.67 ± 0.04	0.53 ± 0.07	0.44 ± 0.04	0.57 ± 0.06	0.59 ± 0.05
C14:1	0.09 ± 0.04	0.04 ± 0.00	0.03 ± 0.01	0.03 ± 0.00	0.03 ± 0.00	0.04 ± 0.01	0.04 ± 0.01
C15:0	1.79 ± 0.04	1.79 ± 0.05	1.20 ± 0.02	1.51 ± 0.16	0.94 ± 0.02	1.29 ± 0.09	2.02 ± 0.11
C15:1	0.06 ± 0.01	0.03 ± 0.01	0.14 ± 0.05	0.08 ± 0.00	0.05 ± 0.01	0.04 ± 0.01	0.03 ± 0.00
C16:0	21.19 ± 0.46	20.87 ± 0.30	19.62 ± 0.23	18.04 ± 1.41	14.31 ± 0.06	17.84 ± 0.87	18.75 ± 1.27
C16:1	0.61 ± 0.03	0.35 ± 0.00	0.38 ± 0.03	0.33 ± 0.05	0.24 ± 0.04	1.13 ± 0.13	0.25 ± 0.02
C17:0	1.46 ± 0.06	0.85 ± 0.00	0.97 ± 0.06	1.05 ± 0.03	0.36 ± 0.01	0.40 ± 0.03	0.41 ± 0.05
C18:0	9.58 ± 0.89	3.98 ± 0.10	3.52 ± 0.15	3.07 ± 0.06	4.36 ± 0.19	4.59 ± 0.08	11.26 ± 0.13
C18:1n9	21.21 ± 0.12	9.52 ± 0.29	7.89 ± 0.22	8.67 ± 0.30	18.30 ± 0.33	22.62 ± 0.31	27.20 ± 0.15
C18:2n6	32.28 ± 0.60	59.19 ± 0.17	62.41 ± 0.66	63.34 ± 1.43	58.46 ± 0.73	48.57 ± 1.46	34.61 ± 0.99
C18:3n3	1.08 ± 0.19	0.20 ± 0.03	1.16 ± 0.11	1.03 ± 0.03	0.20 ± 0.03	0.24 ± 0.04	1.63 ± 0.03
Lipophilic compounds							
C20:0	0.53 ± 0.05	0.12 ± 0.01	0.12 ± 0.01	0.09 ± 0.01	0.15 ± 0.01	0.18 ± 0.00	0.35 ± 0.04
C20:1	0.20 ± 0.02	0.09 ± 0.00	0.08 ± 0.02	0.08 ± 0.00	0.10 ± 0.02	0.08 ± 0.01	0.06 ± 0.01
C20:2	0.18 ± 0.00	0.14 ± 0.00	0.15 ± 0.01	0.16 ± 0.02	0.19 ± 0.03	0.19 ± 0.02	0.13 ± 0.02
C20:3n3 + C21:0	0.21 ± 0.03	0.08 ± 0.01	0.10 ± 0.01	0.15 ± 0.01	0.18 ± 0.01	0.17 ± 0.01	0.08 ± 0.01
C20:5n3	nd	nd	nd	0.19 ± 0.03	0.18 ± 0.02	nd	nd
C22:0	1.16 ± 0.17	0.10 ± 0.01	0.25 ± 0.03	0.38 ± 0.09	0.33 ± 0.02	0.40 ± 0.07	0.75 ± 0.09
C22:1n9	1.90 ± 0.11	0.26 ± 0.00	0.23 ± 0.04	0.16 ± 0.08	0.20 ± 0.02	0.30 ± 0.02	0.16 ± 0.04
C23:0	1.72 ± 0.24	0.09 ± 0.00	0.09 ± 0.02	0.18 ± 0.05	0.08 ± 0.00	0.10 ± 0.00	0.07 ± 0.02
C24:0	3.03 ± 0.02	0.94 ± 0.09	0.59 ± 0.06	0.68 ± 0.02	0.69 ± 0.02	1.04 ± 0.02	1.33 ± 0.15
Total SFA (% of total FA)	42.19 ± 0.16 ^a^	30.10 ± 0.05 ^c^	27.43 ± 0.05 ^cd^	25.78 ± 0.15 ^d^	21.87 ± 0.03 ^e^	26.62 ± 0.09 ^d^	35.82 ± 0.15 ^b^
Total MUFA (% of total FA)	24.07 ± 0.07 ^b^	10.28 ± 0.06 ^d^	8.75 ± 0.06 ^d^	9.36 ± 0.07 ^d^	18.92 ± 0.07 ^c^	24.21 ± 0.08 ^b^	27.74 ± 0.20 ^a^
Total PUFA (% of total FA)	33.75 ± 0.20 ^d^	59.62 ± 0.06 ^b^	63.82 ± 0.26 ^ab^	64.87 ± 0.30 ^a^	59.21 ± 0.16 ^b^	49.17 ± 0.38 ^c^	36.44 ± 0.26 ^d^

Nd—not detected. Caproic acid (C6:0); Caprylic acid (C8:0); Capric acid (C10:0); Lauric acid (C12:0); Myristic acid (C14:0); Myristoleic acid (C14:1); Pentadecanoic acid (C15:0); *cis*-10-Pentadecenoic acid (C15:1); Palmitic acid (C16:0); Palmitoleic acid (C16:1); Heptadecanoic acid (C17:0); Stearic acid (C18:0); Oleic acid (C18:1n9); 12- Linoleic acid (C18:2n6); α-Linolenic acid (C18:3n3); Arachidic acid (C20:0); Eicosenoic acid (C20:1); *cis*-11,14-Eicosadienoic acid (C20:2); *cis*-11,14,17-Eicosatrienoic acid and Heneicosanoic acid (C20:3n3 + C21:0); *cis*-5,8,11,14,17-Eicosapentaenoic acid (C20:5n3); Behenic acid (C22:0); Erucic acid (C22:1n9); Tricosanoic acid (C23:0); Lignoceric acid (C24:0). In each row, different letters mean significant differences between species (*p* < 0.05).

**Table 2:** Composition in hydrophilic compounds of wild and cultivated mushrooms of Ghana (mean ± SD).

	*Pleurotus tuber-regium*	*Termitomyces robustus*	*Lentinus squarrosulus* strain SQW	*Lentinus squarrosulus* strain LSF	*Pleurotus ostreatus* strain EM-1	*Pleurotus sajor-caju* strain PScW	*Auricularia auricula*
Free sugars (g/100 g dw)							
Fructose	nd	nd	nd	0.55 ± 0.15 ^a^	0.30 ± 0.09 ^b^	0.32 ± 0.01 ^b^	nd
Mannitol	0.35 ± 0.00 ^e^	4.71 ± 0.16 ^a^	0.70 ± 0.03 ^d^	1.43 ± 0.22 ^c^	0.87 ± 0.02 ^d^	1.99 ± 0.07 ^b^	0.68 ± 0.01 ^d^
Sucrose	nd	nd	nd	nd	nd	nd	0.67 ± 0.02
Trehalose	1.50 ± 0.03 ^f^	9.92 ± 0.34 ^c^	11.09 ± 0.20 ^b^	12.76 ± 0.34 ^a^	12.74 ± 0.13 ^a^	6.61 ± 0.10 ^d^	2.52 ± 0.01 ^e^
Total sugars	1.85 ± 0.03 ^a^	14.63 ± 0.50 ^a^	11.79 ± 0.22 ^c^	14.74 ± 0.71 ^a^	13.91 ± 0.05 ^b^	8.92 ± 0.18 ^d^	3.87 ± 0.03 ^e^
Organic acids (g/100 g dw)							
Oxalic acid	0.69 ± 0.00 ^a^	0.33 ± 0.01 ^b^	0.17 ± 0.01 ^e^	0.30 ± 0.00 ^c^	0.29 ± 0.01 ^cd^	0.32 ± 0.00 ^b^	0.27 ± 0.02 ^d^
Fumaric acid	0.01 ± 0.01 ^d^	0.16 ± 0.01 ^a^	0.09 ± 0.01 ^c^	0.13 ± 0.02 ^b^	0.18 ± 0.02 ^a^	0.18 ± 0.00 ^a^	0.01 ± 0.01 ^d^
Total organic acids	0.70 ± 0.01 ^a^	0.49 ± 0.00 ^b^	0.26 ± 0.01 ^d^	0.43 ± 0.02 ^c^	0.47 ± 0.03 ^b^	0.50 ± 0.00 ^b^	0.28 ± 0.03 ^d^
Phenolic acids and cinnamic acid (mg/100 g dw)							
*p*-Hydroxybenzoic acid	0.08 ± 0.00 ^g^	2.43 ± 0.02 ^b^	4.46 ± 0.04 ^a^	1.40 ± 0.03 ^d^	1.56 ± 0.01 ^c^	0.43 ± 0.03 ^f^	1.09 ± 0.02 ^e^
*p*-Coumaric acid	nd	0.24 ± 0.01 ^a^	0.21 ± 0.01 ^b^	nd	0.21 ± 0.01 ^b^	nd	nd
Total phenolic acids	0.08 ± 0.02 ^g^	2.67 ± 0.03 ^b^	4.67 ± 0.05 ^a^	1.40 ± 0.03 ^d^	1.77 ± 0.02 ^c^	0.43 ± 0.03 ^f^	1.09 ± 0.02 ^e^
Cinnamic acid	nd	8.06 ± 0.01 ^a^	0.57 ± 0.01 ^d^	0.79 ± 0.01 ^c^	1.82 ± 0.01 ^b^	0.13±0.01 ^e^	0.10±0.01 ^f^

Nd—not detected. In each row, different letters mean significant differences between species (*p* < 0.05).

Reis *et al.* [21] also described the presence of fructose, mannitol and trehalose in a commercial sample of *P. ostreatus*, but in much lower amount than the one found in this work. Gupta *et al.* [22] also found the same free sugars (fructose, mannitol and trehalose) in a sample of *P. sajor-caju* cultivated on wheat straw supplemented with raw and detoxified mahua cake. Beluhan and Ranogajec [18] reported the presence of mannitol, trehalose, mannose and glucose in a wild sample of *P. ostreatus* from Croatia.

Oxalic and fumaric acids were the organic acids found in the studied mushroom species, with the prevalence of oxalic acid in all the samples. The profile of organic acids described by Barros *et al.* [23] for a commercial sample of *P. ostreatus* was slightly different since the authors also detected malic and citric acids.

*p*-Hydroxybenzoic, *p*-coumaric and cinnamic acids were also found in the studied species; *p*-hydroxybenzoic acid was found in all the samples, while *p*-coumaric acid was only detected in *T. robustus*, *L. squarrosulus* strain SQW and *P. ostreatus*. Cinnamic acid was found in almost all samples, with the exception of *P. tuber-regium*. Nevertheless, Reis *et al.* [24] described the presence of protocatechuic acid, in addition to the three ones previously mentioned, in a commercial sample of *P. ostreatus*. The levels of *p*-hydroxybenzoic acid described in this study were similar to the ones herein reported.

### 2.3. Minerals

The variations and mean concentrations of ten macro- (sodium, potassium, calcium, phosphorus and magnesium) (Table 3) and micro- elements (iron, zinc, copper and manganese), and the heavy metal lead (Table 3) were examined in the wild and cultivated species of the mushrooms under study. The samples were shown to be rich in potassium. The trend in decreasing order of macro elements content for the studied mushrooms was K > P ~ Na > Mg > Ca.

Living organisms require varying amounts of “heavy metals”. Iron, cobalt, copper, manganese, molybdenum, and zinc are required by humans, but excessive levels can be damaging to the organism. Other heavy metals such as mercury, plutonium, and lead are toxic metals and their accumulation over time in the bodies of animals can cause serious illnesses.

Copper (Cu) as stated is an essential metal, which serve as a constituent of some metalloenzymes, and is required in haemoglobin synthesis and catalysis of metabolic growth [25]. The average concentration of Cu in the studied samples was 0.05 mg/kg, which was far below the safe limit of 40 mg/kg set by WHO [26]. Copper levels in mushrooms reported in literature are 4.71–51.0 mg/kg [27], 13.4–50.6 mg/kg [28], and 12–181 mg/kg [29]. These values recorded for wild mushrooms correspond to samples collected near industrial sites.

Iron (Fe) is an essential metal involved in biochemical processes. The average Fe content recorded in the studied wild and cultivated mushrooms was 2.33 mg/kg and 0.44 mg/kg, respectively, which are below the safe limit of 15 mg/kg set by WHO. Levels of Fe reported in literature were 146–835 mg/kg [29], 31.3–1190 mg/kg [30], and 180–407 mg/kg [31].

Manganese (Mn) is an essential metal needed for biological systems such as metalloproteins [32]. The lowest and the highest level of Mn present in the studied mushrooms were 0.02 ± 7.07 × 10^−5^ mg/kg and 0.04 ± 0.09 × 10^−1^ mg/kg, respectively, which are also below the toxicity limit of 400–1000 mg/kg.

**Table 3:** Composition in macro- and micro-elements of wild and cultivated mushrooms of Ghana (mean ± SD).

		**Concentrations of Macro Elements (mg/kg dw)**				
Source	Mushroom species	Na	K	P	Ca	Mg
Wild	*Pleurotus* *tuber-regium*	5.00 ± 1.41 × 10^−4^ ^b^	7.46 ± 7.07 × 10^−3^ ^f^	4.46 ± 7.07 × 10^−4^ ^c^	0.34 ± 0.01 × 10^−1^ ^e^	0.90 ± 0.01 × 10^−1^ ^g^
	*Termitomyces robustus*	2.90 ± 0.01 ^d^	20.30 ± 0.07 ^a^	4.05 ± 7.07 × 10^−4^ ^e^	0.40 ± 1.40 × 10^−4^ ^d^	0.72 ± 7.78 × 10^−4^ ^f^
Cultivated	*Lentinus squarrosulus* strain SQW	5.00 ± 7.07 × 10^−4^ ^b^	11.66 ± 7.07 × 10^−3^ ^d^	5.37 ± 0.01 ^b^	0.47 ± 0.04 × 10^−1^ ^c^	1.42 ± 0.07 × 10^−1^ ^b^
	*Lentinus squarrosulus* strain LSF	3.65 ± 0.07 ^c^	15.40 ± 0.28 ^c^	4.44 ± 2.82 × 10^−4^ ^d^	1.57 ± 0.06 ^a^	1.48 ± 0.08 × 10^−1^ ^a^
	*Pleurotus ostreatus* strain EM-1	3.80 ± 0.01 ^c^	17.40 ± 0.01 ^b^	2.11 ± 7.07 × 10^−5^ ^f^	0.39 ± 0.02 ^de^	1.21 ± 0.04 × 10^−1^ ^d^
	*P. sajor-caju* strain PScW	15.00 ± 7.07 × 10^−5^ ^a^	15.00 ± 0.01 ^c^	11.34 ± 2.80 × 10^−4^ ^a^	0.20 ± 0.00 ^f^	1.32 ± 0.01 ^c^
	*Auricularia auricula*	2.00 ± 7.07 × 10^−5^ ^e^	10.60 ± 0.01 ^e^	1.39 ± 7.07 × 10^−4^ ^g^	0.55 ± 0.08 × 10^−1^ ^b^	1.09 ± 0.01 ^e^
		**Concentrations of Micro Elements (mg/kg dw)**				**Concentrations of Heavy Metal (mg/kg dw)**
		Fe	Mn	Zn	Cu	Pb
Wild	*Pleurotus* *tuber-regium*	2.40 ± 7.07 × 10^−4^ ^a^	0.06 ± 7.07 × 10^−4^ ^b^	0.13 ± 2.10 × 10^−3 c^	0.04 ± 7.07 × 10^−4^ ^c^	0.13 ± 7.78 × 10^−4^ ^e^
	*Termitomyces robustus*	2.24 ± 0.02 × 10^−1^ ^b^	0.07 ± 7.07 × 10^−4^ ^a^	0.15 ± 0.01 ^c^	0.05 ± 1.41 × 10^−4^ ^c^	0.61 ± 0.03 × 10^−1^ ^a^
Cultivated	*Lentinus squarrosulus* strain SQW	0.61 ± 0.01 ^c^	0.02 ± 0.04 × 10^−1^ ^d^	0.20 ± 7.07 × 10^−3^ ^b^	0.02 × 10^−1^±3.54 × 10^−4^ ^d^	0.07 ± 0.04 × 10^−1^ ^f^
	*Lentinus squarrosulus* strain LSF	0.56 ± 0.02 ^d^	0.04 ± 0.09 × 10^−1^ ^c^	0.15 ± 0.03 ^c^	0.08 ± 7.07 × 10^−3^ ^a^	0.41 ± 0.06 × 10^−1^ ^c^
	*Pleurotus ostreatus* strain EM-1	0.40 ± 0.03 ^e^	0.03 ± 0.05 × 10^−1^ ^de^	0.33 ± 2.80 × 10^−3^ ^a^	0.06 ± 8.49 × 10^−3^ ^b^	0.40 ± 0.04 × 10^−1^ ^d^
	*P. sajor-caju* strain PScW	0.60 ± 0.01 ^c^	0.02 ± 7.07 × 10^−5^ ^e^	0.07 ± 1.41 × 10^−4^ ^d^	0.02 × 10^−1 ^± 7.07 × 10^−4^ ^d^	0.07 ± 0.01 ^g^
	*Auricularia auricula*	0.37 ± 0.06 × 10^−1^ ^e^	0.04 ± 0.01 × 10^−1^ ^cd^	0.12 ± 0.01 ^c^	0.07 ± 7.07 × 10^−4^ ^ab^	0.60 ± 0.01 ^b^

Na—sodium; K—potassium; P—phosphorus; Ca—calcium; Mg—magnesium; Fe—iron; Mn—manganese; Zn—zinc; Cu—copper; Pb—lead. In each column, different letters mean significant differences between species (*p* < 0.05).

The literature has reported levels of Mn as 14.2–69.7 mg/kg [28], 12.9–93.3 mg/kg [29], and 14.5–63.6 mg/kg [31]. According to Vonugopal and Lucky [33], lead (Pb) is toxic even at trace levels and the impairment related to lead toxicity in humans include abnormal size and haemoglobin content of the erythrocytes, hyperstimulation of erythropoisis and inhibition of haem synthesis. The maximum level of Pb present in the studied wild mushrooms was 0.60 ± 0.00 mg/kg, which is far below the 10.0 mg/kg limit set by the WHO [26]. Lead levels reported in literature are 0.75–7.77 mg/kg [27], 1.43–4.17 mg/kg [29], and 0.40–2.80 mg/kg [34].

Zinc (Zn) is an essential metal and a component of a wide variety of different enzymes in which it is involved in catalytic, structural and regulatory roles. The minimum and maximum levels of Zn present in the studied wild and cultivated samples were below the permissible limit of 60 mg/kg of Zn in foods [26]. Zn levels reported in literature were 45.2–173.8 mg/kg [28], 33.5–89.5 mg/kg [29], and 29.3–158 mg/kg [31]. The trend in decreasing order of elements present in wild and cultivated samples was Fe > Pb > Zn > Mn > Cu and Fe > Pb > Zn > Cu > Mn, respectively. All these values were statistically lower than the acceptable safe limits set by the WHO.

The lower values recorded in this study as compared to other studies can be attributed to various factors such as the analytical methods used and the substrate on which they were cultivated. Mushrooms are known to possess a very effective mechanism that enables them to readily take up some heavy metals from the ecosystem [35]. The accumulation of heavy metals in mushrooms seems to be affected by environmental and fungal related factors. Environmental factors such as organic matter content, pH and metal concentration in soil and fungal factors such as the species, morphological part of the fruit body, developmental stages, age of mycelium, intervals between fructifications and biochemical composition [36].

### 2.4. Antioxidant Activity

Five *in vitro* assays were used to evaluate the antioxidant properties of the samples: Folin-Ciocalteu assay, ferricyanide/Prussian blue, scavenging effects on DPPH radicals, inhibition of β-carotene bleaching and inhibition of lipid peroxidation in brain cell homogenates, as shown in Table 4. The results obtained indicate that all the samples tested exhibit some antioxidant potential. Globally, *A. auricula* gave the highest reducing power (measured by ferricyanide/Prussian blue assay) and DPPH radical scavenging activity. Nevertheless, *L. squarrosulus* strain SQW showed the highest reducing power (measured by the Folin-Ciocalteu assay), β-carotene bleaching inhibition and lipid peroxidation activity in TBARS assay. A reducing power of 281.15 AAE mg/g has been recorded for ethanolic extracts of wild *L. squarrosulus* using the phosphomolybdenum (PMo) assay [37]. It should be highlighted that *P. ostreatus* gave higher antioxidant activity than a commercial sample studied by Reis *et al.* [24].

Among the two *L. squarrosulus* strains studied, strain SQW had a significantly higher antioxidant activity than strain LSF according to both the Folin-Ciocalteu reagent assay and the TBARS assay. On the contrary, a significantly lower antioxidant activity was recorded for strain SQW than strain LSF according to the DPPH radical scavenging activity assay.

**Table 4:** Antioxidant activity of wild and cultivated mushrooms of Ghana (mean ± SD).

		*Pleurotus tuber-regium*	*Termitomyces robustus*	*Lentinus squarrosulus* strain SQW	*Lentinus squarrosulus* strain LSF	*Pleurotus ostreatus* strain EM-1	*Pleurotus sajor-caju* strain PScW	*Auricularia auricula*
Reducing power	Folin-Ciocalteu (mg GAE/g extract)	11.08 ± 01 ^e^	26.68 ± 0.71 ^bc^	49.04 ± 1.06 ^a^	26.47 ± 0.27 ^bc^	17.66 ± 0.69 ^d^	25.47 ± 0.03 ^c^	28.12 ± 0.75 ^b^
	Ferricyanide/Prussian blue (EC_50_; mg/mL)	2.06 ± 0.03 ^a^	1.24 ± 0.01 ^d^	1.14 ± 0.01 ^e^	1.15 ± 0.01 ^e^	1.64 ± 0.03 ^b^	1.35 ± 0.01 ^c^	0.70 ± 0.01 ^f^
Radical scavenging activity	DPPH radical scavenging activity (EC_50_; mg/mL)	17.39 ± 0.23 ^a^	4.78 ± 0.05 ^d^	5.33 ± 0.27 ^c^	3.68 ± 0.10 ^e^	5.28 ± 0.17 ^c^	9.52 ± 0.15 ^b^	2.01 ± 0.09 ^f^
	β-carotene/linoleate (EC_50_; mg/mL)	3.79 ± 0.46 ^a^	1.76 ± 0.17 ^c^	0.97 ± 0.05 ^d^	0.80 ± 0.08 ^d^	1.59 ± 0.07 ^c^	2.48 ± 0.29 ^b^	1.76 ± 0.09 ^c^
Lipid peroxidation inhibition	TBARS (EC_50_; mg/mL)	1.04 ± 0.05 ^b^	0.43 ± 0.04 ^d^	0.18 ± 0.01 ^e^	1.00 ± 0.05 ^b^	0.15 ± 0.01 ^e^	0.75 ± 0.07 ^c^	1.51 ± 0.15 ^a^

Concerning the Folin-Ciocalteu assay. Higher values mean higher reducing power; for the other assays. The results are presented in EC_50_ values. That means, higher values correspond to lower reducing power or antioxidant potential. EC_50_: Extract concentration corresponding to 50% of antioxidant activity or 0.5 of absorbance for the Ferricyanide/Prussian blue assay. In each row, different letters mean significant differences between species (*p* < 0.05).

*Pleurotus tuber-regium* showed the lowest antioxidant activity based on all the assays used with the exception of the TBARS assay, which indicated that the species antioxidant activity was higher than that of *A. auricula* but comparable to that of *L. squarrosulus* strain LSF. These results indicate that the *in vitro* assays used in determining the antioxidant activity of a given sample could give a biased impression about the antioxidant potential of the sample, as different groups of compounds present in the extract possibly react differently to the various reactive oxygen species used in the assays.

Ferreira *et al.* [38] have demonstrated that antioxidant potential of caps of Portuguese *Lactarius deliciosus* (L.) Gray and *Tricholoma portentosum* (Fr.) Quél. are higher than that of the stipes. It will therefore be interesting to determine the portion of the fruiting body of the mushrooms studied herein with the highest nutritional composition and antioxidant potential. This information will be useful for mushroom consumers and cultivators, who will be able to make informed decisions about the part of the mushrooms they should consume/sell more and for industries, which would be interested in utilizing the antioxidants in mushrooms for various applications.

## 3. Experimental Section

### 3.1. Mushroom Species

The mushroom species used in this study are presented in Table 5. The wild samples were dried in a fabricated field dryer for 6 h, whilst the cultivated samples were sun dried for 2 days. All the samples were further pulverized and stored in the freezer until transported to Mountain Research Centre (CIMO), ESA, Polytechnic Institute of Bragança, Portugal for chemical composition analysis (unless minerals analysis) and antioxidant activity evaluation.

**Table 5:** Information about the studied mushroom species.

Mushroom Species	Common Name	Edibility	Habitat
*Pleurotus* *tuber-regium* (Fr.) Singer	King of oyster	Edible	Picked from the forest (wild)
*Termitomyces robustus* (Beeli) R. Heim	Termite mushroom	Edible	Picked from the forest (wild)
*Lentinus squarrosulus* Mont. strain SQW	Unknown	Edible	Cultivated on mixed sawdust of *Triplochiton scleroxylon* and *Chlorophora excelsa*
*Lentinus squarrosulus* Mont. strain LSF	Unknown	Edible	Cultivated on mixed sawdust of *Triplochiton scleroxylon* and *Chlorophora excelsa*
*Pleurotus sajor-caju* (Fr.) Singer strain PScW	Grey oyster mushroom	Edible	Cultivated on mixed sawdust of *Triplochiton scleroxylon* and *Chlorophora excelsa*
*Pleurotus ostreatus* (Jacq.) P. Kumm strain EM-1	Oyster mushroom	Edible	Cultivated on mixed sawdust of *Triplochiton scleroxylon* and *Chlorophora excelsa*
*Auricularia auricula* (L.) Underw	Wood ear mushroom	Edible	Cultivated on mixed sawdust of *Triplochiton scleroxylon* and *Chlorophora excelsa*

### 3.2. Standards and Reagents

Acetonitrile 99.9% was of HPLC grade from Fisher Scientific (Lisbon, Portugal). The fatty acids methyl ester (FAME) reference standard mixture 37 (standard 47885-U) was purchased from Sigma (St. Louis, MO, USA), as were other individual fatty acid isomers, sugars (d(-)-fructose, d(-)-mannitol, d(+)-raffinose pentahydrate, and d(+)-trehalose), phenolic compounds (gallic, *p*-hydroxybenzoic, *p*-coumaric, protocatechuic and cinnamic acids) and Trolox (6-hydroxy-2,5,7,8-tetramethylchroman-2-carboxylic acid). 2,2-Diphenyl-1-picrylhydrazyl (DPPH) was obtained from Alfa Aesar (Ward Hill, MA, USA). All other chemicals and solvents were of analytical grade and purchased from common sources. Water was treated in a Milli-Q water purification system (TGI Pure Water Systems, Greenville, SC, USA).

### 3.3. Spawn and Substrate Preparation

Tissue culture preparations of five cultivated mushroom species were inoculated onto Malt Extract Agar for mycelia growth of 7 days. Spawn was then prepared on sorghum grains in accordance to Oei [39]. The spawns were inoculated on sawdust of a mixture of *Triplochiton scleroxylon* and *Chlorophora excelsa* using the plastic bag method [40,41]. Fruit bodies of these mushrooms were harvested after the first flush, afterwards for each harvested mushroom sample, a mixture was performed and replicates were picked from these and previously sun dried samples, as stated above.

### 3.4. Nutritional and Energetic Value

The samples were analyzed for chemical composition (protein, fat, carbohydrates and ash) using the AOAC procedures [42]. The samples crude protein content (N × 4.38) was estimated by the macro-Kjeldahl method; the crude fat was determined using a Soxhlet apparatus by extracting a known weight of sample with petroleum ether; the ash content was determined by incineration at 600 ± 15 °C. Total carbohydrates were calculated by difference and total energy was calculated according to the following equations: Energy (kcal) = 4 × (g protein + g carbohydrates) + 9 × (g fat).

### 3.5. Lipophilic Compounds: Fatty Acids

Fatty acids were determined after a transesterification procedure as described previously by the authors [2,43]. The fatty acids profile was analyzed with a DANI 1000 gas chromatographer (GC) equipped with a split/splitless injector and a flame ionization detector (FID). Fatty acid identification was made by comparing the relative retention times of FAME peaks from samples with standards. The results were recorded and processed using Clarity 4.0.1.7 Software (DataApex, Podohradska, Czech Republic) and expressed in relative percentage of each fatty acid.

### 3.6. Hydrophilic Compounds

*Free Sugars.* Sugars were determined by a high performance liquid chromatograph (HPLC) system consisted of an integrated system with a pump (Smartline system 1000, Knauer, Berlin, Germany), degasser system (Smart line manager 5000) and an auto-sampler (AS-2057 Jasco, Easton, MD, USA), coupled to a refraction index detector (RI detector Knauer Smartline 2300) as previously described by the authors [2,43]. Sugars identification was made by comparing the relative retention times of sample peaks with standards. Data were analyzed using Clarity 2.4 Software. Quantification was based on the RI signal response of each standard, using the internal standard (IS, raffinose) method and by using calibration curves obtained from the commercial standards of each compound. The results were expressed in g per 100 g of dry weight.

*Organic acids*. Organic acids were determined following a procedure previously described by the authors [23]. The analysis was performed using a Shimadzu 20A series UFLC (Shimadzu Coperation, Kyoto, Japan). Detection was carried out in a diode array detector (DAD), using 215 nm and 245 nm (for ascorbic acid) as preferred wavelengths. The organic acids found were quantified by comparison of the area of their peaks recorded at 215 nm with calibration curves obtained from commercial standards of each compound. The results were expressed in g per 100 g of dry weight.

*Phenolic acids and related compound*. Phenolic acids determination was performed using a Shimadzu 20A series ultra fast liquid chromatograph (UFLC, Shimadzu Cooperation, equipment described above) as previously described by Stojković *et al.* [43]. The phenolic compounds were characterised according to their UV, mass spectra and retention times compared with commercial standards when available. For the quantitative analysis of phenolic compounds, a calibration curve was obtained by injection of known concentrations (5–80 μg/mL) of different standard compounds. The results were expressed in mg per 100 g of dry weight.

### 3.7. Minerals

The whole mushrooms (pileus and stipe) were used for chemical analysis. All the minerals Copper (Cu), Iron (Fe), Manganese (Mn), Magnesium (Mg), Lead (Pb), Zinc (Zn), Calcium (Ca) were analyzed by AAS (PerkinElmer AAnalyst 400, Waltham, MA, USA) spectrophotometer (Jenway 6300, Staffordshire, UK) after wet digestion of the samples [40]. The ascorbic acid method for color development was used in the determination of Phosphorus (P). A flame photometer (Jenway PFP 7, Staffordshire, UK) was used in the determination of Potassium (K) and Sodium (Na).

### 3.8. Antioxidant Activity Evaluation

*Extract preparation.* The lyophilized samples (1 g) were extracted twice by stirring with methanol (40 mL) for 1 h and subsequently filtered through Whatman No. 4 paper. The combined methanolic extracts were evaporated at 40 °C (rotary evaporator Büchi R-210, Büchi, Flawil, Switzerland) to dryness and re-dissolved in methanol (20 mg/mL). Successive dilutions were made from the stock solution and submitted to the following *in vitro* assays.

*Antioxidant activity assays.* Reducing power was evaluated by the reduction of the Folin-Ciocalteu reagent at 765 nm (Analytik Jena spectrophotometer; Jena, Germany), and by the capacity to convert Fe^3+^ into Fe^2+^ at 690 nm using a microplate reader (ELX800 Bio-Tek Instruments, Inc; Winooski, VT, USA). DPPH radical scavenging activity was evaluated in the microplate reader mentioned above, measured at 515 nm. Inhibition of β-carotene bleaching was evaluated through the β-carotene/linoleate assay, measured at 470 nm. Lipid peroxidation inhibition in porcine brain homogenates was evaluated by the decrease in thiobarbituric acid reactive substances (TBARS), measured at 532 nm [43]. The sample concentrations (mg/mL) providing 50% of antioxidant activity or 0.5 of absorbance (EC_50_) were calculated from the graphs of antioxidant activity percentages (DPPH, β-carotene/linoleate and TBARS assays) or absorbance at 690 nm (ferricyanide/Prussian blue assay) against sample concentrations. In the Folin-Ciocalteu assay the results were expressed as mg of gallic acid equivalents (GAE) per g of extract. Trolox was used as a positive control.

### 3.9. Statistical Analysis

Three samples were used and all the assays were carried out in triplicate. The results are expressed as mean values and standard deviation (SD). The results were analysed using one-way analysis of variance (ANOVA) followed by Tukey’s HSD Test with *p* = 0.05. This analysis was carried out using SPSS v. 22.0 program (IBM Corp., Armonk, NY, USA).

## 4. Conclusions

Oyster mushrooms (*Pleurotus* species) are the main cultivated mushrooms in Ghana, and studies on their cultivation using different agricultural wastes have been performed. Nevertheless, the knowledge about nutritional value, chemical composition in lipophilic and hydrophilic molecules, minerals and antioxidant properties of mushroom species from Ghana, are not yet well disseminated. This research concluded that either wild or cultivated samples are nutritionally rich in carbohydrates and proteins, presenting low levels of macro- and micro-elements, and low fat contents (making them excellent for inclusion in low caloric diets). With respect to their high antioxidant potential, mushrooms can find different applications, namely as functional foods or a source of nutraceuticals, maintaining and promoting health and life quality.
